# Urban-rural disparities in skilled birth attendance among women in Ethiopia: Multivariate decomposition analysis

**DOI:** 10.1371/journal.pone.0327565

**Published:** 2025-07-08

**Authors:** Tsegereda Abebe Andargie, Emebet Birhanu Lealem, Betelhem Abebe Andargie

**Affiliations:** 1 International Institute for Primary Health Care- Ethiopia, Addis Ababa, Ethiopia; 2 Department of Epidemiology and biostatistics, Institute of public Health, College of Medicine and Health Sciences, University of Gondar, Gondar, Ethiopia; University of Georgia College of Public Health, UNITED STATES OF AMERICA

## Abstract

**Introduction:**

Skilled birth attendants play an important role in reducing maternal mortality. Although Ethiopia has shown a remarkable reduction in maternal mortality, maternal health service utilization, such as skilled birth attendance, remains low. Thus, this study aims to assess the urban-rural disparity in skilled birth attendance in Ethiopia using the 2019 Ethiopian mini demographic health survey.

**Methods and materials:**

The study was based on data obtained from demographic and health surveys in Ethiopia. A total weighted sample of 5,527 women who gave birth within 5 years preceding the survey was included. The result of descriptive statistics was reported using the frequency, percentages, graphs, and tables. A multivariate decomposition analysis was used to identify factors contributing to the disparity of skilled birth attendance across residence. Statistical significance was defined at a 95% confidence interval with a p-value of less than 0.05.

**Result:**

Skilled birth attendance utilization among women in Ethiopia was 49.8% (95% CI: 48.5–51.1). The disparity in skilled birth attendance coverage between urban and rural areas was significantly high (Urban coverage was 72.1% and rural coverage was 42.5%). Endowment coefficients (women’s characteristics) explained 88% of the urban-rural disparity in the magnitude of skilled birth attendance. Women with secondary and above educational status, four or more antenatal care visits, households with televisions and radio, women in the richest wealth index and women with five or more parity were the determinants that explained the urban-rural disparity in skilled birth attendance.

**Conclusion and recommendations:**

There was a significant disparity in skilled birth attendance utilization between urban and rural areas. Factors like maternal education, wealth status, antenatal care visits, and media access explained the disparity. To attain equitable progress towards universal coverage of SBA, special efforts and resources should be targeted towards rural women. Initiatives aimed at enhancing access to health services and health care consultations for the rural community are also recommended.

## Introduction

Maternal mortality is an essential indicator to assess social and economic growth and effectiveness of a country’s healthcare system [1]. According to the World Health Organization, in 2020, more than 287 000 women worldwide lost their lives during pregnancy or after giving birth. From these, 95% of all maternal deaths that year occurred in low- and middle-income countries [2]. Given this evidence, maternal mortality remains a public health issue and the Sustainable Development Goals (SDGs) aim to reduce the global maternal mortality ratio to fewer than 70 deaths per 100,000 live births by 2030 [3].

In Ethiopia, even though progress is being made to improve maternal health, maternal mortality is still high with 412 deaths per 100,000 live births as reported in the recent national demographic health survey [4]. Maternal mortality in Ethiopia is a complex problem with different underlying causes and lack of access to high-quality maternal healthcare is one of them. Many Ethiopian women face this issue due to inadequate infrastructure, shortage of skilled medical professionals, and limited availability of essential maternal health services like skilled birth attendance (SBA), antenatal care(ANC), and emergency obstetric care [5,6]. According to a study conducted in Ethiopia, only 15.6% of mothers received SBA at home or at a healthcare facility. Rural women faced a disadvantage, with just 4.5% receiving SBA, compared to 64.1% of their urban counterparts [7].

One of the key indicators used to monitor maternal morbidity and mortality is the proportions of births attended by skilled health personnel (skilled birth attendant). According to the World health organization (WHO), a skilled birth attendant is “an accredited health professional - such as a midwife, doctor or nurse - who has been educated and trained to proficiency in the skills needed to manage normal (i.e. uncomplicated) pregnancies, childbirth and the immediate postnatal period, and in the identification, management and referral of women and neonates for complications” [8]. SBA is the most essential approach in reducing maternal mortality and obstetric complications because the majority of maternal deaths and obstetric complications occur at the time of delivery and may not be anticipated beforehand [9]. In 2020, 83% of births worldwide were assisted by a skilled birth attendant [10,11] and in Ethiopia, skilled birth attendants assisted 50% of births, increasing from 26% in 2016 to 48% in 2019, with a magnitude of 40% in rural areas and 70% in urban areas [4].

Various studies indicate that factors such as maternal education [12], ANC visits [12], means of transportation [13], Spousal support [13], and complications in preceding pregnancy [13] contribute to the urban-rural disparity in skilled birth attendance.

Ethiopia has made significant efforts over the past few decades to enhance its health-care system and improve maternal health. The country is one of the few African countries that have met its goal of increasing maternal health and lowering child mortality [14]. Furthermore, the Ethiopian health sector transformation plan (HSTP-I), targeted to reach 90% SBA coverage by 2020 and based on the estimation of 2019 Ethiopian Mini demographic health survey, the national Skill birth attendant coverage is expected to reach to 94% by 2025 [4,9].

Despite the nation’s efforts to promote SBA, home delivery with traditional birth attendants remains high, especially among hard-to-reach rural areas [15]. Unfortunately, traditional birth attendants are also responsible for using potentially harmful cultural practices or for delaying seeking assistance from healthcare professionals in case of complications or emergencies [16,17]. Furthermore, key mother and child health services were negatively impacted by the disparate distribution of maternal health services within and across regions, as well as among population subgroups due to a range of Sociodemographic factors [18].

Assessing urban-rural discrepancies in SBA among Ethiopian women is crucial due to significant healthcare inequities, as rural areas have lower SBA utilization. This approach allows understanding factors contributing to these disparities, which helps in identifying key policy interventions to improve maternal health in Ethiopia. Moreover, the study will be able to support policy makers to use the evidence and design appropriate intervention to avert disparities in using SBA service in the country. Therefore, we aimed to assess the urban-rural disparity of utilizing skilled birth attendant services and identify the determinants of skilled birth attendant utilization using 2019 Mini DHS data in Ethiopia

## Method and materials

### Data source, study setting and study period

The study was done using the 2019 Ethiopian Mini demographic and health survey (EMDHS). Ethiopian demographic health survey is a comprehensive and nationally representative cross sectional survey conducted in Ethiopia as a part of the worldwide DHS program every five years interval since 2000. The 2019 EMDHS is the fifth DHS implemented in Ethiopia [4]. At the time of the survey, Ethiopia was subdivided into nine regional states and two administrative cities. Each region is divided into zones and zones into administrative units called woredas and each woreda is classified into the simplest administrative unit called kebeles. The data collection period of the survey was March 21, 2019, to June 28, 2019 [4].

### Source and study population

The source population for this study was all reproductive aged (15–49) women who gave birth within 5 years prior to the 2019 EMDHS in Ethiopia**,** Whereas the study population was all reproductive aged women (15–49) who gave birth within 5 years prior each survey in the selected enumeration areas in Ethiopia.

### Sample size determination

The detailed sample size determination of the study can be found in the 2019 EMDHS report [19]. For this study individual women’s record (IR file) were used. Sample weights were calculated in IR dataset of EMDHS and missing values of the outcome were excluded based on the DHS guideline. Consequently, this study included a total weighted sample size of 5,527 women who gave birth 5 years prior to survey.

### Sampling procedure

A Stratified clustered two stages sampling was used to select the respondents The 2019 Ethiopia Population and Housing Census (EPHC) served as the sampling frame for the 2019 EMDHS, providing the basis for selecting survey participants.

Each region was stratified into urban and rural areas, yielding 21 sampling strata. Then Samples of enumeration area (EA) were selected independently in each stratum in two stages. The first stage used in each survey being the EAs consisting of urban and rural areas. The second stage used in the survey is households with an equal probability systematic selection. The 2019 EMDHS had a cluster size of 305. A representative sample of 9,150 households from 93 urban clusters and 212 rural clusters was selected, with 30 households from each cluster. Of the selected households, interviews were successfully conducted with 8,663 households. Accordingly, 9,012 eligible women were selected for the interview and were completed with 8,885 women [4].

### Outcome of interest

The outcome variable of this study was whether women assisted by skilled professionals in their recent childbirth or not. The outcome was coded as a binary variable (“yes” = 1, if women were assisted by doctors, nurses/midwives, health officers, or health extension workers and “no” = 0, otherwise) [15].

### Stratifying variable

Place of residence was the key independent variable used to stratify women by their skilled birth attendance and was categorized as rural (coded as “0”) and urban (coded as “1”).

### Independent variables

After searching literature, socio-demographic and maternal characteristics were considered as independent variables [19–21]. These included maternal age, women’s educational status, wealth status, marital status, sex of the household head, household size, household with television, household with radio, region, antenatal care visits, place of delivery and parity.

### Data collection procedure

The questionnaire for EMDHS was adopted from the Measure DHS project to reflect the health issues in Ethiopia. The EMDHS used the household, men’s, women’s Questionnaire, Anthropometry Questionnaire, the Health Facility Questionnaire, and the Field Workers Questionnaire. The DHS questionnaire was first prepared in English then translated to three major languages Amharic, Afaan Oromo, and Tigrigna. EMDHS’s data collectors were recruited based on language skills, academic qualifications and previous survey experience and trained for collection. The data were collected through interviewing techniques and fieldwork procedures. Electronic materials like tablets were also used to collect the response of the respondent.

### Data management and analysis

Data were analyzed using STATA version 17.0. Weighted frequencies and percentages were calculated to account for DHS and design effects. The result of descriptive statistics was reported as frequency, percentages, a graph, and tables. Pearson’s chi-square test was used to assess the association between SBA and the independent variables.

Decomposition analysis was employed to identify the contributing factors to the disparity in Skilled birth attendance across urban and rural residents.The decomposition model additively explains the difference in Skilled birth attendance among women living in urban and rural by **Endowments** (i.e. disparity in Skilled birth attendance is due to the difference in the selected variables of women between urban-rural residents, which is explained) and **Coefficients** (i.e. disparity in Skilled birth attendance is due to the effects of those selected variable, that are unexplained). Multivariate decomposition technique determines the high outcome group (urban women in this study) automatically and uses the low outcome group (rural women in this study) as a reference category. This analysis was used over other methods for decomposition analysis because it gives detailed decomposition and standard errors for both the characteristics component and the coefficient component. [23]. Variables with a *p*-value less than 0.25 from the bi-variable decomposition analysis were selected as candidate variable for multivariable decomposition analysis. Finally, Statistical significance of association in the decomposition analysis was declared at a p-value of <0.05.

### Ethical consideration

EMDHS were conducted after obtaining ethical clearance from Ethiopia Health and Nutrition Research Institute Review Board, the Ministry of Science and Technology and Institutional Review Board of ICF International. Written informed consent was obtained from all women before data collection and all data were anonymized from individual’s names, addresses and other identifiers. Approval for the use of data was sought and received from the DHS program https://www.dhsprogram.com/. We confirm that the study was conducted according to the Declaration of Helsinki.

## Result

### Characteristics of study participants

A total weighted of 5,527 women who gave birth in five years prior to each survey were included in this study. More than half (61%) of rural residents and 32% of urban residents had no education. The majority of residents were married, with 92% in urban areas and 95% in rural areas.. More than three-fourths of households had male heads: 78% in urban areas and 89% in rural areas. Regarding the wealth index distribution, almost 60% of the urban residents were in the richest category, while only 4% rural residents were in the richest group. About 44% of the study participants from urban areas and one-third (37% of women from rural areas had four or more access to antenatal care services. Home delivery is still higher among rural residents (60%), while only 30% of urban residents delivered at home. The chi-square test result showed that all included characteristics varied significantly across urban and rural areas with a p-value of <0.001(**Table 1**).

**Table 1:** Characteristics of the participants in Ethiopia, Mini-EDHS 2019.

Characteristics	Frequency (%)		Chi-square(P-value)
	Urban(1367)	Rural (4160)	
Maternal age 15-19 20-24 25-29 30-34 ≥35	56 (4) 271(20) 456 (34) 309(21) 275(21)	207(6) 761(18) 1,300(31) 884(21) 1,008(24)	0.001
Marital status Not in union Married/living with partner	114(8) 1,253(92)	188(5) 3,972(95)	<0.001
**Sex of Household head** **Male** **Female**	1,068(78) 299(22)	3,708(89) 452(11)	**<0.001**
**Maternal Educational status** No education Primary Secondary and above	443(32) 562(41) 362(27)	2,518(61) 1,395(34) 247(5)	**<0.001**
**Wealth index** Poorest poorer Middle Richer Richest	121(9) 74(5) 82(6) 259(20) 831(60)	1,200(29) 1,124(27) 961(23) 701(17) 174(4)	**<0.001**
**Household size** **<6** **≥6**	947(69) 420(31)	2523(61) 1637(39)	**<0.001**
**Have television** **Yes** **No**	689(50) 678(50)	113(7) 4,047(93)	**<0.001**
**Have radio** **Yes** **No**	496(36) 571(64)	900(22) 3,260(78)	**<0.001**
**Region** Tigray Afar Amhara Oromia Somalia Benishangul-Gumuz SNNPRs Gambela Harari Addis Ababa Dire-Dawa	106(8) 23(2) 227(17) 388(28) 121(9) 20(1) 292(21) 13(1) 7(0.5) 156(11) 14(1)	265(6) 63 (2) 823 (20) 1,823 (44) 288(7) 47(1) 814(19) 12 (0.28) 10(0.23) 0 (0) 16(0.38)	**<0.001**
**Number of ANC visit** No visit 1-3 visit ≥4 visit	502(37) 262(19) 603(44)	852(29) 963(34) 1,085(37)	**<0.001**
**Parity** 1 2-4 ≥5	281(20) 726(53) 360(27)	545 (13) 1,845(44) 1,770(43)	**<0.001**
**Place of delivery** Home Facility	405(30) 962(70)	2,495(60) 1,665(40)	**<0.001**

### Urban-rural disparity in skilled birth attendance in Ethiopia

**Fig 1:**
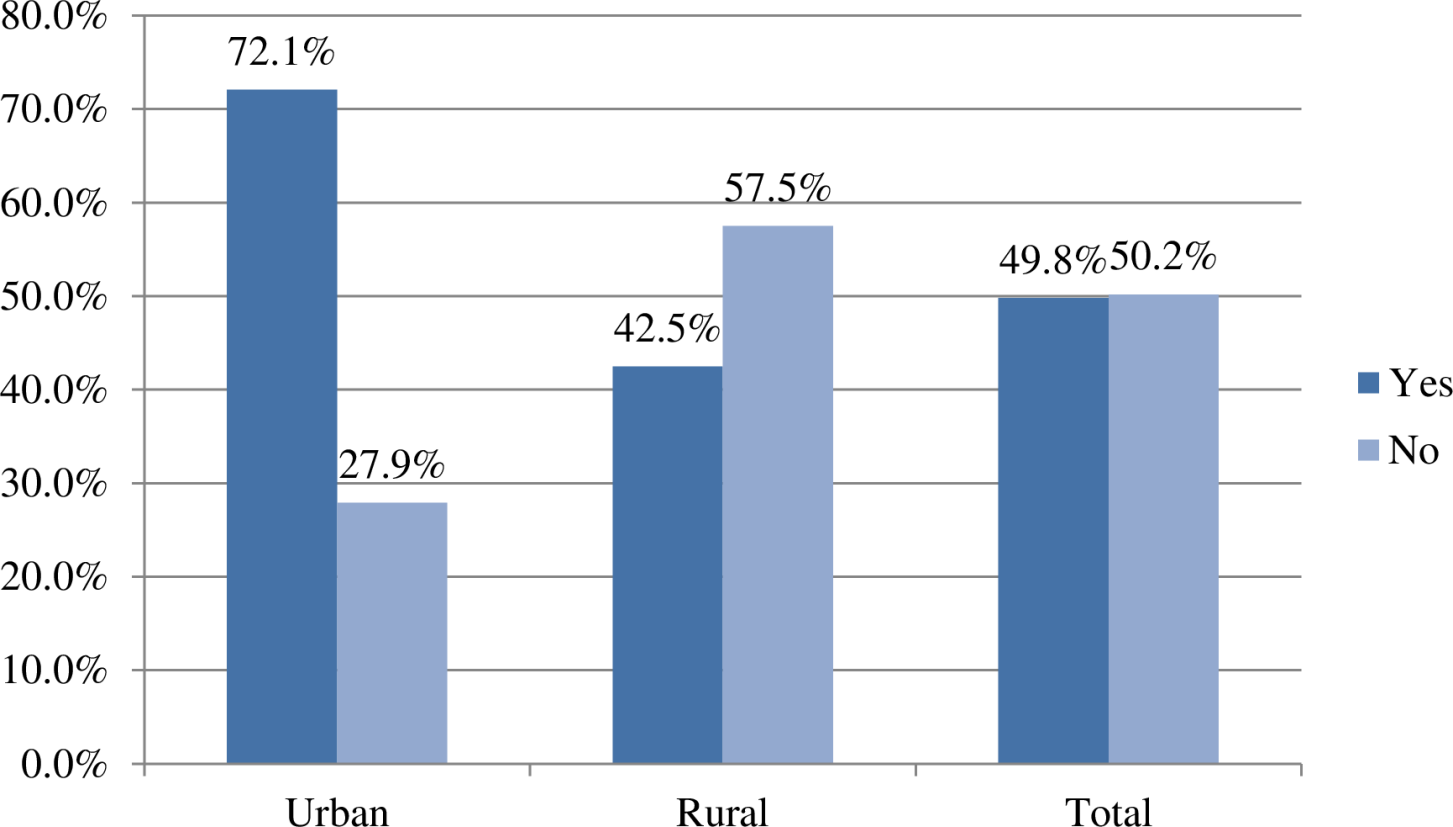
Urban and rural disparities of skilled birth attendance among women in Ethiopia, using EMDHS 2019.

The proportion of SBA among women was 49.8% (95% CI: 48.5–51.1). Utilization of SBA in urban residents was higher than that in rural residents with 72.1% and 42.5%, respectively (Fig 1).

### Residential skilled birth attendance by participants’ characteristics

Skilled birth attendance coverage varied by women’s characteristics in the two contexts. Nearly three-fourth (71%) of women aged 35 and older in urban areas had their birth attended by skilled professionals while only 37% of women had skilled birth attendance in rural areas. Moreover, the majority (91%) of women residing in urban areas with skilled birth attendance had secondary and above educational status, only 60% of rural women with skilled birth attendance had a similar education level. Except for marital status and household sex, all variables were significantly associated with skilled birth attendance in both urban and rural residential **(**Table 2**).**

**Table 2:** Residential distribution of skilled birth attendance among women in Ethiopia, Mini EDHS 2019.

Characteristics	Skilled birth attendance (%)			
	Urban (N = 985 )	Chi-square (p-value)	Rural(N = 1,768)	Chi-square (p-value)
**Maternal age** 15-19 20-24 25-29 30-34 ≥35	34(61) 203(75) 343(75) 211(68) 194(71)	12.8(0.012)	121(58) 394(52) 526(40) 351(40) 376(37)	26.0(<0.001)
**Marital status** Not in union Married/living with partner	74(65) 911(73)	1.77(0.182)	84(45) 1684(42)	3.2(0.071)
**Household head sex** **Male** **Female**	765(72) 220(74)	0.16(0.693)	1592(43) 176(39)	1.58(0.208)
**Maternal Educational status** No education Primary Secondary and above	246(55) 409(73) 329(91)	157.1(<0.001)	769(32) 773(55) 198(60)	401.6(<0.001)
**Wealth index** Poorest poorer Middle Richer Richest	40(33) 45(67) 24(30) 147(57) 729(88)	227.8<0.001)	252(21) 441(39) 471(49) 459(66) 146(83)	617.7(<0.001)
**Household size** <6 ≥6	1,225(49) 543(33)	79.1(<0.001)	732(77) 253(60)	35.7(<0.001)
**Household has television** Yes No	95(84) 1673(41)	106(<0.001)	616(89) 369(54)	126((<0.001)
**Household has radio** Yes No	427(52) 1295(40)	58(<0.001)	409(83) 576(66)	43.5(<0.001)
**Region** Tigray Afar Amhara Oromia Somalia Benishangul-Gumuz SNNPRs Gambela Harari Addis Ababa Dire-Dawa	106(100) 15(65) 196(86) 222(57) 67(55) 17(81) 183(63) 12(92) 6(92) 150(96) 13(95)	255(<0.001)	166(63) 12(18) 390(47) 748(41) 39(14) 27(58) 371(46) 5(46) 5(46) 0 8(49)	422(<0.001)
**Number of ANC visit** No visit 1-3 visit ≥4 visit	264(53) 194(74) 527(88)	166(<0.001)	109(13) 488(51) 755(70)	703(<0.001)
**Parity** 1 2-4 ≥5	369(68) 815(44) 584(323)	162.9(<0.001)	249(89) 543(75) 193(54)	91.5(<0.001)

### Factors contributing to the urban-rural disparity in skilled birth attendance

There was a 29.6% disparity in SBA coverage between women residing in urban and rural areas, with women in urban areas most likely to have a SBA (p-value<0.001). The result showed that this difference in SBA between urban and rural women was significantly explained by the endowment component or women’s characteristics only, which accounted for 88% (β = 0.2279, 95%CI: [0.1352, 0.3206]) of the disparity (Table 3).

**Table 3:** Overall decomposition analysis result of urban–rural disparity among women in Ethiopia, 2019 EMDHS.

Skilled birth attendance	Coefficient	P-value	95% confidence interval (CI)	Percentage (pct.)
E	0.2279	<0.001	[0.1352,0.3206]	88**
C	0.0320	0.580	[-0.0814, 0.1454]	12
R	0.2599	<0.001	[0.21044, 0.30946]	

E: component represents change in characteristics; C: component represents change in Coefficient R: Residual;**: p-value<0.001.

Table 4 shows the results of the detailed decomposition analysis. Holding the effects of coefficients constant, the difference in the proportion of women with secondary and above educational status accounted for 5.8% of the urban-rural disparity in skilled birth attendance. Meaning that if the composition of women with secondary and above education was equalized the gap in SBA would be narrowed by 5.8%. ANC visits played a significant role in explaining the urban-rural gap in SBA. The difference in the proportion of women with four or more ANC visits accounted for 23.9% of the gap. This means that if rural women had the same level of ANC utilization as urban women, the SBA disparity would be reduced by nearly one-fourth. Additionally, wealth status was a major factor in the disparity. The difference in the proportion of women in the richest wealth quintile explained 30.7% of the urban-rural gap in SBA. Access to media sources also contributed to the disparity. The difference in television ownership explained 11.7%, while radio ownership accounted for 2.1% of the gap. Finally, number of children influenced the gap, with the difference in the proportion of women with five or more children explaining 19.2% of the urban-rural disparity.

**Table 4:** Detailed decomposition analysis result of urban–rural disparity among women in Ethiopia, 2019 EMDHS.

Variables	Difference due to characteristics				Difference due to coefficient			
	Coeff (95% CI)			Pct.	Coeff (95% CI)		Pct.	
Maternal age								
15-19	1				1			
20-24	-0.00666[-.007392,0.007378]			-0.025	0.010787[-0.0519,0.07354]			4.6
25-29	0.0007031[-0.00413, 0.00554]			0.27	0.039014 [-0.063534, 0.14156]			15.0
30-34	-0.00014[-0.00072, 0.000428]			-0.05	0.024406 [-0.049949,0.098761]			9.4
≥35	-0.01225[-0.03544,0.01094]			-4.71	0.051086 [-0.04962,0.15179]			19.7
Maternal Educational status								
No education	1				1			
Primary	0.00171[-0.00518,0.008622]			0.7	-0.014397[-0.05958,0.03078]		-5.5	
Secondary and above	0.014962 [0.01626,0.046191]			5.8*	-0.006505[0.02057,0.00756]		-2.5	
Wealth status								
Poorest			1		1			
Poorer			0.00807[-0.044986 ,0.061135]	3.1	-0.033628[-0.10005 ,0.03279]		-12.9	
Middle			0.01302 [-0.031784,0.057839]	5.0	-0.0426[-0.10874 ,0.023541]		-16.4	
Richer			-0.00154[-0.00483, 0.0045308]	0.06	-0.03311[-0.0895,0.023314]		-12.7	
Richest			0.079847[0.054214,0.21391]	30.7*	-0.007193[-0.02360,0.009215]		-2.8	
Marital status								
Not in union	1				1			
Married /living with partner	-0.00621[-0.02149 ,0.009058]			-2.4	0.055731[-0.14947,0.26093]		21.4	
Sex of household head								
Male	1				1			
Female	0.009569 [-0.004259,0.023399]			3.7	0.00774[-0.0091913,0.024675]		2.9	
Household size								
<6	1				1			
≥6	-0.002991[-.021292,0.01531]			-1.2	0.018442[-0.040268,0.077152]		7.1	
Has television								
Yes	0.030402[0.05950,0.1203]			11.7*	-0.00139[-0.009376,0.0065941]		-0.5	
No	1				1			
Has radio								
Yes	0.0055581[0.015278,0.026395]			2.1*	0.010392[-0.024486,0.045269]		3.9	
No	1				1			
ANC visit								
No visit		1				1		
1-3		-0.0213[-0.03402,0.0087107]		-8.2		-0.009399[-0.05665,0.037851]	-3.6	
≥4		0.062305 [0.038225,0.086385]		23.9*		-0.018227[-0.06996 ,0.033506]	7.0	
Parity								
1		1				1		
2-4		-0.013149[-0.02583, -0.000463]		-5.1		-0.03506[-0.11891,0.048782]	-13.4	
≥5		0.049866[0.0056447,0.094088]		19.2*		-0.060663 [-0.17148,0.050156]	-23.4	

## Discussion

The study aimed to assess the urban-rural disparities in SBA among Ethiopian women using the 2019 EMDHS. The finding of this study demonstrated that there was a statistically significant difference in SBA among women in urban and rural areas. Nearly half of births (49.8%) were attended by skilled professionals. Women living in urban areas accounted for nearly two-third (72.5%) of the SBA, while the rest 42.4% were among rural participants. In this study, the disparity in SBA magnitude between urban and rural areas was significantly attributed only to the change in the endowment characteristics. Maternal educational status, ANC visit, household with televisions and radio, wealth index and parity were the determinants of the urban-rural disparity in SBA.

SBA coverage was found to be 29.6% higher among mothers in urban areas than in rural areas. Similarly, studies conducted in Ethiopia, Ghana and Nigeria indicated that there was a disparity in the number of births attended by SBA between rural and urban women [22–24]. In addition to these similar studies from low and middle-income nations such as Nigeria and India showed comparable results [13,25,26]. In Nigeria, the study found that women living in rural regions experience significant barriers to receive SBA due to long distances to health facilities, poor road networks, and insufficient transportation options [13] Similarly, in India, a study found that women in urban areas benefit from improved healthcare services and higher educational levels, which leads to increased SBA utilization [25,26].

This can be explained by women living in urban areas generally having better access to healthcare services and skilled birth attendants, which allows timely professional care during delivery. Moreover, maternal health awareness and education are higher in urban settings, resulting in increased utilization of SBA [27]. In addition to this, urban families often have higher financial resources, allowing them to cover transportation and other health care related expenses. Urban areas also benefit from more developed infrastructure and transportation networks, making health care services more accessible [28,29]. Furthermore, rural areas frequently rely on traditional birth attendants, largely due to limited access to support systems that promote the use of SBA. These combined factors magnify the urban-rural discrepancy in SBA coverage among Ethiopian women [30].

The results also revealed that the difference in proportion of women who had secondary education or more indicated the gap in SBA coverage between urban and rural by 5.8%, keeping the coefficients constant. This finding is also supported by studies done in Ethiopia and other parts of Africa [31,32]. This could be because educated women have better capacity and understanding to make informed decisions about their health because they are aware of complications associated with pregnancy and accessible health care services, and they are more likely to be financially secure than those who are uneducated [33,34].

The findings of this study showed that when the coefficient effect is held constant, the difference in proportion of women who had four or more ANC visits explained the gap in SBA coverage between urban and rural areas by 23.9%. This finding is also supported by studies done in Ethiopia [7,35,36]. This might be explained by the fact that during ANC follow-up, women can get guidance on birth preparedness, including selecting a qualified birth attendant and preparing for anticipated complications. Furthermore, regular meetings with healthcare providers give mothers a chance to learn more about SBA and its advantages. As a result, mothers feel more confident and encouraged to use skilled birth attendance [35].

Our finding showed that the difference in proportion of women in richest wealth quintiles explained the gap in SBA coverage between urban and rural areas by 30.7%, by keeping the coefficient effect constant. This finding is supported by studies done in Ethiopia and other developing countries [36–38]. This disparity could be attributed to the fact that, while skilled delivery services are provided for free in Ethiopia, there may be extra expenses such as transportation, accommodation and payments for additional supplies required during the delivery process that women in lower and middle quintiles cannot provide. This has a direct impact on whether a woman can really use skilled birth attendance. Women in richest wealth quintiles, on the other hand, can afford these additional expenses, resulting in increased use of skilled birth attendance [39,40].

The observed findings of the study underscored the coefficient effect is held constant, the difference in proportion of households with television and radio explained the gap in SBA coverage between urban and rural areas, by 11.7% and 2.1%. This finding can be supported by studies done in India and Ethiopia [41,42]. This might be attributed to the fact that television and radio have considerably empowered women by encouraging the use of health care services such as skilled birth attendance, which ensures safer delivery. Furthermore, media advocacy plays an important role in raising awareness about the benefits of SBA and birth readiness; therefore, it provides valuable information that enables women to make informed pregnancy decisions. [43,44].

The finding of this study also indicated keeping the coefficients constant; the difference in proportion of women with five or more parity explained the gap in SBA coverage between urban and rural areas by 19.2%. This might be due to the reason that women with higher parity tend to be more aware of the potential complications during childbirth due to their prior experiences. This awareness drives them to seek professional healthcare to mitigate possible risks of complications. Additionally, through their previous pregnancies, these women have likely developed a trust in the healthcare system and familiarity with healthcare providers. This accumulated trust and experience make them more inclined to recognize the vital role that skilled birth attendants play in ensuring the safety of both mother and child during delivery. Therefore, this increases the likelihood of preferring skilled birth attendance. Moreover, interaction of parity with other factors such as education is crucial in understanding its full impact on SBA utilization. Education plays a significant role in shaping reproductive behavior and health-seeking decisions. Women with higher education levels are more likely to delay childbearing and limit family size (lower parity). Additionally, educated women are better equipped to understand health information, recognize pregnancy-related danger signs, and utilize the benefits of skilled birth attendance [45].

### Strength and limitation

Though the current study has its own strengths, like it was based on a large dataset representing the whole country, the findings should be interpreted in light of these limitations. Variables which would have explained the urban-rural gaps like women’s media exposure, distance to health facility, health care decision making, maternal occupation, wantedness of pregnancy and health insurance coverage were not included in this study because these variables were not available in EMDHS. These factors likely influence both ANC visits and SBA utilization, potentially changing our estimates. For instance, media exposure raises awareness of maternal health services, while distance and insurance affect access. Future research should address this by using datasets that include these variables. Despite this, our findings provide critical insights into SBA disparities. Besides, since the outcome was based on the self- report, there might be a recall bias or social desirability bias. However, efforts were made to minimize these biases through rigorous data collection procedures.

## Conclusion

The study showed a significant disparity between Urban and rural areas in SBA utilization. The disparity in SBA between urban and rural women was significantly explained by the endowment component or women’s characteristics only. Factors like maternal education, wealth status, ANC visits, parity and household TV and radio explained most of the disparities between urban and rural areas. The findings underscore the need for targeted, equity-focused interventions to improve SBA utilization, especially among rural and socioeconomically disadvantaged populations. Furthermore, integrating these efforts with broader social protection strategies, such as community-based health insurance, may increase SBA utilization among these populations.

### Policy implications

- Expand healthcare facilities in rural areas to provide access to skilled birth attendants. This includes equipping primary health care facilities and increase access to ANC visits in rural regions by introducing mobile health units, especially focusing on reaching women with limited transportation or financial resources. This aligns with Ethiopia’s Health Sector Transformation Plan and National Health Care Financing Strategy as these polices emphasize infrastructure development and community-based health insurance to strengthen primary healthcare services.
- To ensure feasibility, policymakers should consider leveraging existing initiatives that support sustainable facility expansion. Furthermore, prioritizing cost-effective interventions, such as deploying mobile clinics in hard-to-reach areas, could enhance healthcare accessibility without imposing excessive financial strain on the health system. It is recommended that further research on the economic implications of healthcare facility expansion will be essential to guide evidence-based policy decisions.
- Implement targeted educational programs to raise maternal health literacy, especially in rural areas with lower educational achievement.
- Expand media outreach in rural areas through radio and television programs focused on advocating maternal health, SBA benefits, and birth preparedness.

## List of Abbreviations/Acronym:

ANC: Antenatal Care
DHS: Demographic Health Survey
EMDHS: Ethiopian mini–Demographic Health Survey
SBA: Skilled Birth Attendance
SDGs: Sustainable Development Goals
WHO: World Health Organization
